# Supplementary figures and images for: Gene expression in soft-shell clam (Mya arenaria) transmissible cancer reveals survival mechanisms during host infection and seawater transfer
Source: PLoS Genet. 2025 Mar 31;21(3):e1011629. doi: 10.1371/journal.pgen.1011629 (PMC11978232; doi:10.1371/journal.pgen.1011629)

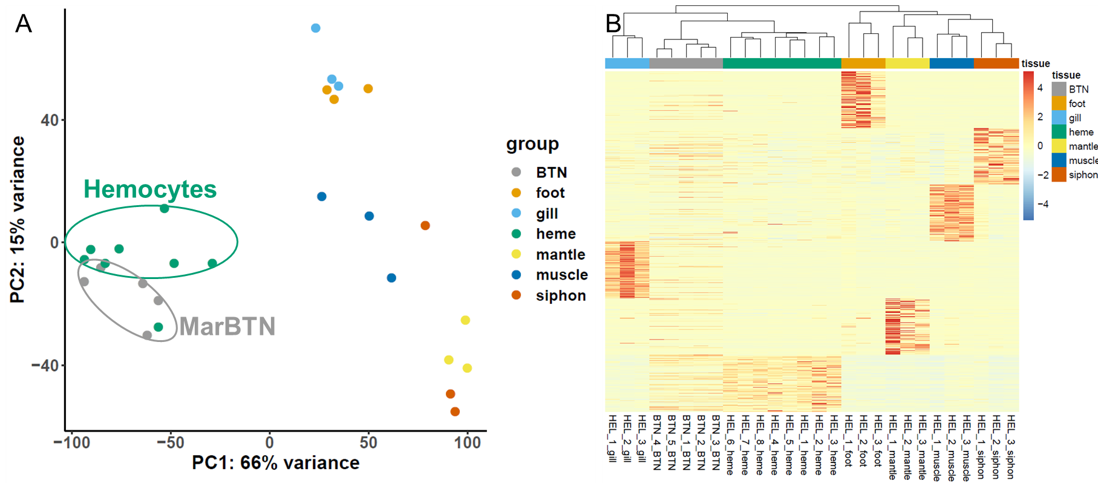

Supplement: S1 Fig — (A) Principal component analysis of normalized expression across all genes, with PC1 separating MarBTN and hemocytes from all other tissues. (B) Hierarchical clustering of all RNA sequenced samples (excluding ASW-treated samples) by the expression of the top 100 most significant genes expressed in each specific healthy tissue relative to all other tissues, with heatmap of normalized relative gene expression for each gene. MarBTN (“BTN”) clusters most closely with hemocytes (“heme”), supporting principal component analysis results. Results for both panels closely match similar analyses with previous genome annotation and a smaller sample set [21]. (TIF) [file pgen.1011629.s001.tif]

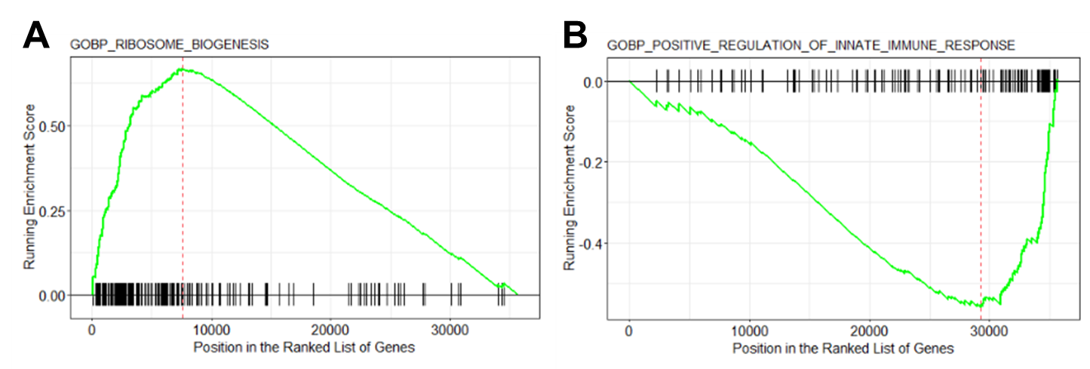

Supplement: S2 Fig — Running enrichment score (green), which increases each time it hits a gene in the gene set (black bars along x-axis) for the ribosome biogenesis biological process (A), one of the top upregulated pathways, and positive regulation of innate immune response biological process (B), one of the top downregulated pathways. Red dotted line marks the peak enrichment score, corresponding to the x-axis of volcano plots. Genes were rank-ordered using “stat” DESeq2 parameter, which corresponds directly to significance ranking, but with positive and negative values for up/down regulated genes. (TIF) [file pgen.1011629.s002.tif]

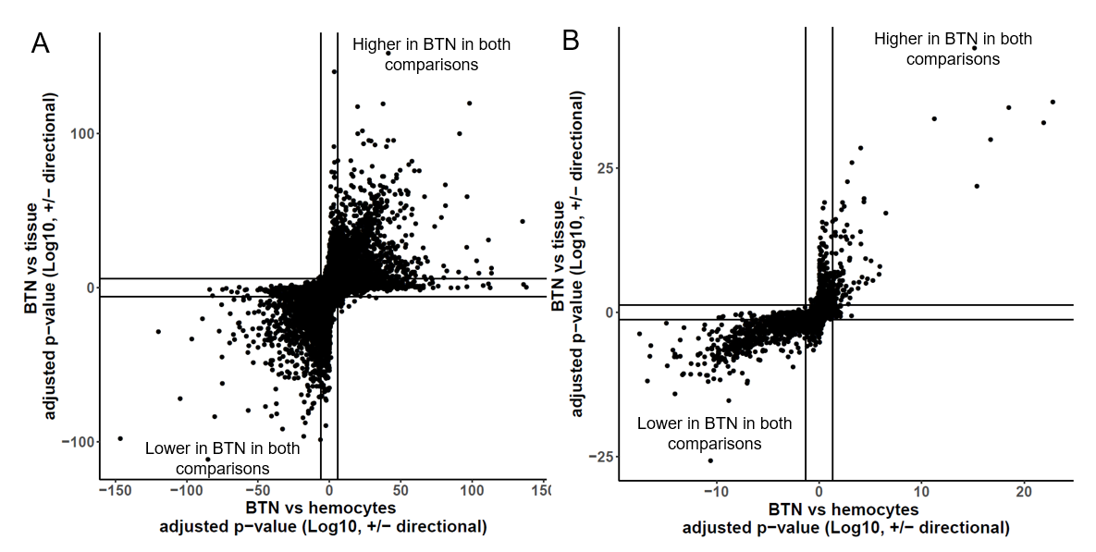

Supplement: S3 Fig — Adjusted p-values, further adjusted to be positive for upregulation and negative for downregulation, from MarBTN versus healthy hemocytes differential expression results (x-axes) and MarBTN versus solid tissues differential expression results (y-axes) for individual genes (A) and gene pathways (B). In general, genes and pathways that are upregulated versus hemocytes are also upregulated versus solid tissues, indicating that major differential expression results and conclusions are not artifacts of the comparison with hemocytes. Lines represent false discovery-corrected p< 0.05 significance thresholds. (TIF) [file pgen.1011629.s003.tif]

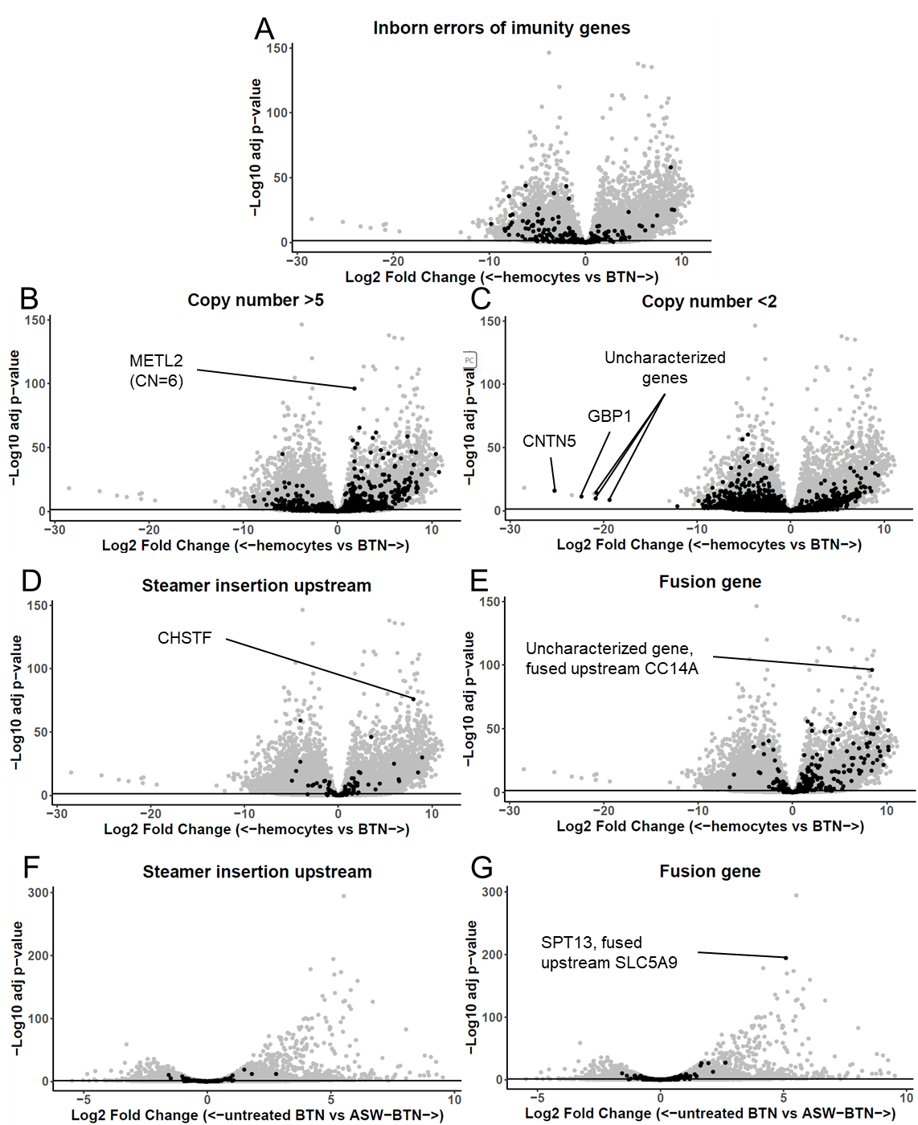

Supplement: S4 Fig — All genes are shown as grey points, and in each panel black points mark those genes known to be associated with human inborn errors of immunity (A), copy number over 5 (B), copy number under 2 (C), Steamer insertions within 2kB upstream (D, F), or gene fusions (E, G). (TIF) [file pgen.1011629.s004.tif]

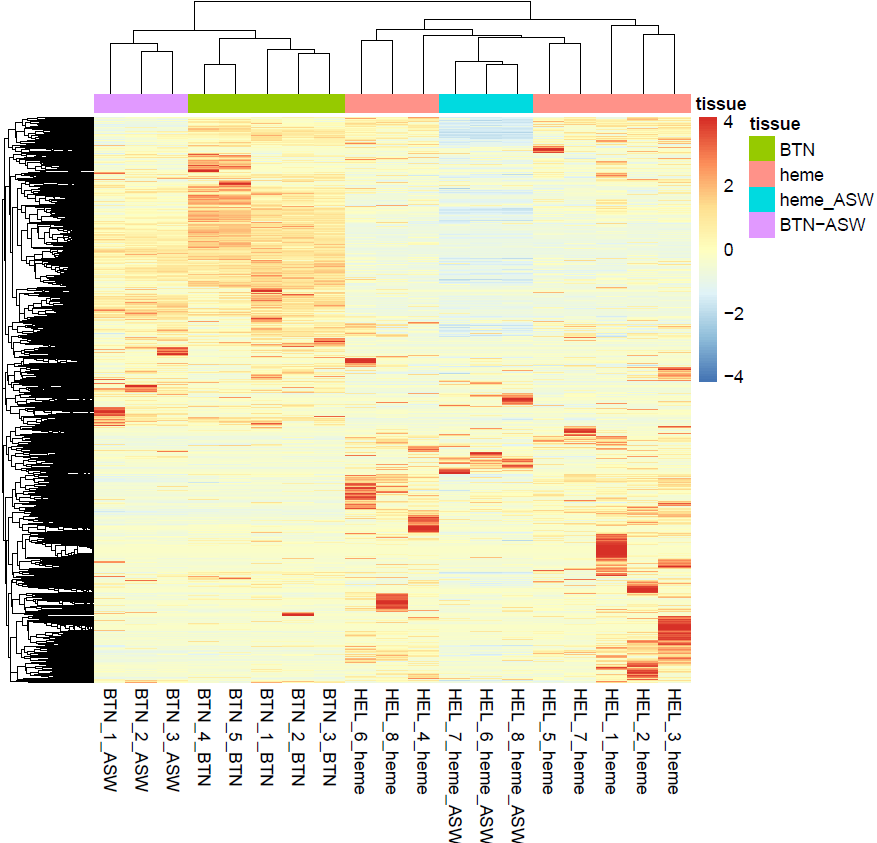

Supplement: S5 Fig — Hierarchical clustering using all expressed genes for untreated healthy hemocytes (“heme” - blue), ASW-treated healthy hemocytes (“heme_ASW” - pink), untreated MarBTN (“BTN” - red) and ASW-treated MarBTN (“BTN-ASW” - green). Samples are labeled by their source clam and treatment. (TIF) [file pgen.1011629.s005.tif]

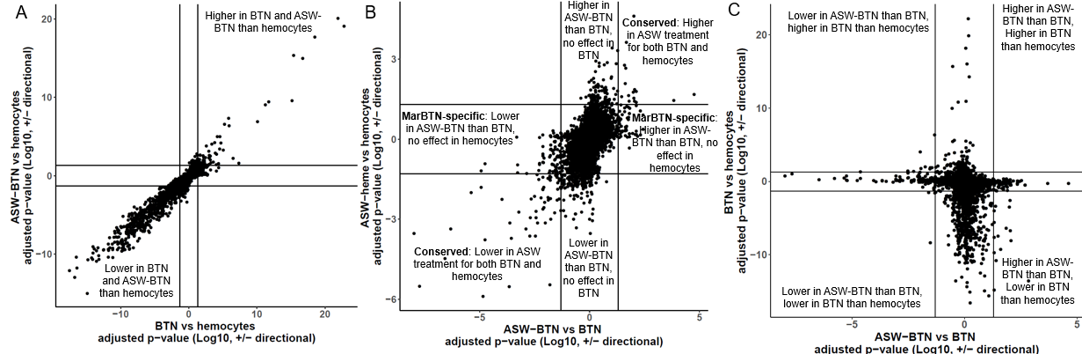

Supplement: S6 Fig — Adjusted p-values, further adjusted to be positive for upregulation and negative for downregulation, to compare gene set enrichment analysis results from (A) ASW-treated MarBTN and untreated MarBTN each versus hemocytes, (B) ASW-treated versus untreated for each of MarBTN and hemocytes, and (C) the BTN response to seawater compared with the initial BTN/hemocytes results. In (A) results are highly correlated, with the same top up- and downregulated pathways regardless of which treatment is compared to healthy hemocytes. In (B), gene set groupings corresponding to conserved and MarBTN-specific seawater responses from Fig 4 are labeled. In (C), very few gene sets exist in the outer quadrants, indicating little overlap between the significant gene sets in the two comparisons. Lines represent false discovery-corrected p< 0.05 significance thresholds. (TIF) [file pgen.1011629.s006.tif]
