# Supplementary material for: Gene expression in soft-shell clam (Mya arenaria) transmissible cancer reveals survival mechanisms during host infection and seawater transfer
Source: PLoS Genet. 2025 Mar 31;21(3):e1011629. doi: 10.1371/journal.pgen.1011629 (PMC11978232; doi:10.1371/journal.pgen.1011629)
Supplement: Attachment — Submitted filename: MarBTN_transcriptome_review_responses_v3.docx [file pgen.1011629.s025.docx]

**PGENETICS-D-24-01028**

**Gene expression in soft-shell clam (Mya arenaria) transmissible cancer reveals survival mechanisms during host infection and seawater transfer**

***Thank you to the reviewers for the time and effort taken to thoroughly assess our manuscript and provide constructive feedback. One theme that was reflected by all reviewers was the need for the genome instability section (Fig 2) to be better integrated with the gene-by-gene results in the rest of the paper. We have addressed this issue, adding a supplementary figure and discussion of individual genes affected by CNV/Steamer/fusions throughout the manuscript, where applicable. Our revised manuscript is much stronger for it. Below, you will find author responses to each comment in bold, with reference to the changes made in the manuscript when applicable, which are highlighted in yellow.***

Reviewers' comments:

Reviewer #1: In nature, there are three types of naturally transmissible cancers; one affects dogs (canine transmissible venereal tumour or CTVT), two affect Tasmanian devils and several affect soft-shell clams. The cell identity of these transmissible cancers is poorly understood, which limits our knowledge of the origin and evolution of these extraordinary tumours. In this paper, Hart et al. characterise the transcriptional signature of Mya arenaria bivalve transmissible neoplasia or BTN. The authors performed RNAseq to compare normal clam hemocytes and BTN samples and found a common core signature, supporting the notion that BTN emerged from hemocytes, but also found a substantial number of differentially expressed genes DEGs by gene set enrichment analysis (GSEA). Notably, they detected downregulation of innate immune pathways in BTN, suggesting a mechanism for immune evasion. The authors found increased copy number variation in BTN, which correlated with changes in expression. Notably, when the transcriptional profiling was repeated in the presence of sea water to mimic the extra-host life cycle of BTN or hemocytes, these same innate immune genes were found specifically upregulated in BTN relative to hemocytes, suggesting a potential function in transmission and/or survival.

The results are interesting and point to an important and potentially opposite, function of innate immune pathways in regulating BTN growth and transmission. Characterising the physiological response of hemocytes and BTN to seawater is also of interest. The results are well presented and for the most part convincing, however there are elements of the paper that could be improved.

1. The study is entirely based on RNAseq and some confirmation of key results by different methods would strengthen the conclusions. For example, qPCR on a selected array of genes, or, when suitable antibodies are available, Western blotting or flow cytometry to detect protein levels.

**Given limited resources for non-model organisms and the high quality/reliability of illumina RNA sequencing, we feel that incorporating these methods are out of the scope of our current manuscript and would not change our conclusions outside of confirming that sequencing is reliable in soft-shell clams.**
2. GSEA is a valuable, but it could be complemented by alternative methods such as Ingenuity Pathway Analysis, or other suitable bioinformatics approaches.

**We agree that an investigation into gene pathways would be an interesting follow-up to this work, though we note that because pathways include more information than unordered gene sets, they require the additional assumption that pathways are ordered the same in our species as in the reference pathways set (e.g. humans and well characterized model organisms) and it would therefore be more sensitive than GSEA to gene annotation discrepancies and differences in biological pathways between organisms. We already present complementary lines of evidence in our paper, namely gene-by-gene analysis and GSEA, supporting our major claims and sufficient for an initial investigation into a non-model system. To further add support through an alternative method to our most important finding, widespread immune downregulation, we have added an additional analysis looking at genes known to be mutated in inborn errors of immunity, finding widespread downregulation of these genes and independently verifying this GSEA result (Supp Fig 4A).**
3. In Supplementary Fig. 4, the “ASW” should be labelled “BTN-ASW” for clarity. In the heatmap, differences between groups are not that clear. Could perhaps an additional clustering based on Log2FC or adjusted p be added?

**We have changed the label the “BTN-ASW” (now in Supp fig 5) and added clustering by genes (y-axis) as well as clustering by sample (x-axis) to order the genes in a more intuitive way to see the basis for the sample clusters.**
4. In Supplementary Tables, the genes included in each GSEA pathway should be shown to enable better comparisons.

**We have added a supplementary table 17 with the gene lists for each gene set.**
5. Did the authors test is sea water induced greater activation of Steamer, or other ERVs? Often, activation of ERVs is detected by nucleic acids sensors triggering innate immune activation.

**We did not see greater activation of Steamer in ASW-treated MarBTN cells versus untreated cells. Though Steamer is much more highly expressed in MarBTN than hemocytes (~80-fold), it is not significantly different between ASW-treated MarBTN and untreated MarBTN. In fact, Steamer is expressed more in untreated MarBTN (~67% higher), though this difference is not significant.**
6. Can the authors map the genomic copy number (Figure 2B) to the genes included in the inflammatory pathways detected by GSEA?

**We added an additional analysis looking at copy number differences on a gene-by-gene basis (Supplementary Fig 4B and C). Indeed, we do see some of the immune-related pathways among those with copy number loss. However, it is difficult to do this analysis on a gene set level, since a single regulatory gene copy number difference could be responsible for widespread expression changes, so we kept this analysis on a gene-by-gene level and looked at immune deficiency genes in a separate analysis (see comment 2 response above).**

Reviewer #2: In this article, the authors quantify and analyse gene expression in a transmissible cancer impacting soft-shell clams. The authors are looking to identify genes and pathways that appear to be central for the parasitic ability of these BTNs, which will allow them to get more insights into the processes that drive a cell to become a transmissible cancer. They perform a well-executed differential expression analysis comparing the transmissible cancer to its supposed tissue of origin – healthy hemocytes. They go further by experimentally subjecting hemocytes and transmissible cancer cells to seawater treatment and observing the responses in terms of genes expressed. Their set up and comparison is useful to distinguish genes whose expression varies in both hemocytes and BTN, from genes that only seem to be up or down regulated specifically in BTN. The authors did a good job of summarizing their findings of genes up or down regulated, and do not overstate their conclusions. Some more precisions on technical aspects and statistics would be appreciated to clarify the methods, as well as a better explanation and annotation of the figures which would permit a quicker understanding of the results and discrimination of what is up and down regulated among an extensive number of genes presented.
Here are the details of what would need to be clarified in order to improve this paper:

In the text:
1- The annotation contains 44,373 gene models (according to supplementary table 1). Could the authors possibly state this number in the text, discuss why this is higher than would be expected, and explain that all gene models (44,373 genes) were used in their DEseq analysis.

**We added this number to the main text results and clarified in the DEseq methods section.**
2- P.3 l.13: Please write out what BUSCOs are as well as a quick explanation in the main text.

**We have added the explanation of the acronym and now state that it measures transcriptome completeness.**
3- P.4 l.14: Here in particular (for TENX and CNTN5) and at a few other places, it is not immediately clear in the text if the genes being mentioned are up or down regulated. Specifying that would be helpful.

**We added a clarification in parentheses when it is not otherwise stated and/or log fold change and pvalue, as requested by another reviewer.**
4- p.5 l.2 “which order ranks genes” – explain what this means/how this is done.

**We clarified that this ranking is by significance of differential expression, clarified the wording, and referenced an example illustration (supp fig 2).**
5- P.6 l.7-17: It is to be commended that the authors are careful with interpretations and raise the possibility that the observation of downregulation of immune genes does not automatically mean that those represent and evolutionary response for the cancer as a strategy to evade hosts response. However, the comparison with a pool of other tissues as explained in the paper does not seem to entirely suffice to be convinced that the differences observed between hemocytes and BTN cells solely reflect mechanisms selected for immune evasion/driving cancer. In particular, have the authors considered the possibility that this cancer could have emerged from hemocytes at a different differentiation stage than the fully mature hemocytes used for comparison, and that the differences in some pathways could reflect those differences in cell state/developmental stage?

**We agree that we are not able to conclusively say that the immune downregulation is an evolutionary mechanism to evade host rejection: comparing MarBTN to other tissues reinforces the argument that the immune downregulation is due to a loss of widely-expressed immune genes and not due to the a loss of immune-cell-specific genes to an immune cell, but this is not a direct test of an evolutionary response. It is possible that a different hemocyte cell state origin of MarBTN could in part explain this decrease, though our test against other tissues highlights that regardless of what tissue you compare MarBTN to, it has much lower immune gene expression, so we believe that an immature immune cell having lower immune cell expression than a non-immune tissue is unlikely. We have added this as another alternative and changed our wording to highlight how the test also makes this alternative unlikely.**
6- P.6 l.22: Please provide a little more detail about the study in MtrBTN2 by Burioli et al. to which the genes and pathways are compared, in particular, was this also a BTN vs hemocytes comparison?

**We added more detail on this study.**
7- P.7 l.2: Specify the degrees of freedom for the chi-squared test.

**Done**
8- P.9 l.4-9: The first part about Steamer insertion and the comparison when it is inserted within genes is clear, but what follows with the more insertions within 2kb is a bit confusing. It is not very clear how the comparison is done and what exactly is being compared to what (median fold change? What are the groups and the null hypothesis?). Because of that, it is not straightforward what the p-values in the text are representing (p=0.038 for which comparison and which null hypothesis? One of the labels in the figure suggests that ** p< 0.005, how does that compare?). Could the relatively low sample size in the control group (17) decrease the robustness of the result? Overall, it would be helpful if this part were clarified as it is for now not clear how it confidently contributes to ruling out the hypothesis that increased expression and Steamer could be linked to chromatin accessibility/pre-existing high expression instead of a true causation.

**We agree these Steamer effect comparisons are a bit difficult to follow. We have updated the wording to add clarity to this comparison when considered alongside the figure and figure legend, which helps to visualize the comparisons described.**
9- P.11 l.24: How many genes/ gene sets were up/down regulated in each case?

**We added the number of genes and genesets here (MarBTN-specific) as well as earlier in the seawater treatment section for the number of conserved genes/genesets.**
10- P.12 l.9 Given all the data that the authors have gathered, it could be nice to go a bit further and be more precise about the genes and pathways that have a “reversal of the downregulation observed in untreated MarBTN cells” – How many? Which ones are they specifically? There is probably a way of having a better visualization of for which of these exact pathways this is the case.

**We now specify the number of pathways and that are downregulated in MarBTN cells vs healthy hemocytes.**
11- P. 13 l8-9. “These responses may reflect metabolic processes that are active in proliferative MarBTN, but not hemocytes, that are shut down in response to seawater exposure”: Could the authors comment on whether these specific genes and pathways are upregulated in MarBTN vs hemocytes in their initial (not seawater) comparisons? This would help to support their hypothesis.

**We have added a third panel (Supp Fig 5C: ASWBTNvsBTN on x-axis, BTNvsHeme on y-axis) looking at this comparison. It appears that one of the ASW-response metabolism sets listed is more active in the untreated comparison of MarBTN than hemocytes, while the other metabolism gene sets are not. We have added a discussion of this to the results section.**
12- P.16 l12: Processed data outputs, i.e. gene tables and pathway tables used to create figures, should be supplied as accompanying source data to improve reproducibility (please could this be provided for data underlying Figures 2B and 2C?).

**We now include a supporting information file with tables of data for all figures.**
13- P.17 l.11-13: How certain is it that the cells adhering to the plate are only host hemocytes? Could this protocol of collecting the non-adherent cells be biasing the cell population? Is there a reference for this?
**BTN cells have been shown to be non-adherent, while hemocytes will adhere to a plate within an hour (see papers below). We only expect to find these two cell types in the hemolymph. It is possible that some cancer cells are pulled down by adherent hemocytes, removing them from the cancer samples, but we are more concerned with purifying hemocytes out of cancer samples than losing a small percentage of cancer cells, and allowing cells to adhere for an hour is a very efficient way to accomplish this. We now explain this rationale and cite these papers in the methods.**

- **Reno, P. W., House, M. & Illingworth, A. Flow cytometric and chromosome analysis of softshell clams, Mya arenaria, with disseminated neoplasia. Journal of Invertebrate Pathology 64, 163–172 (1994).**
- **Giersch, R. M. et al. Survival and Detection of Bivalve Transmissible Neoplasia from the Soft-Shell Clam Mya arenaria (MarBTN) in Seawater. Pathogens 11, 283 (2022).**

14- P.17 l.15: Could antibiotics influence the gene expression response? Is there a way to control for that?

**It is possible that the antibiotics could influence gene expression. However, this experiment can’t be done without antibiotics, because clam hemolymph has microbes/parasites that grow up in less than 24hr and are likely to affect the experiment (see Giersch et al. 2022). Our control for this possibility is that both MarBTN and hemocytes have the same antibiotics in their 24hr treatment, so if there is an effect on expression, we expect it to affect both groups similarly.**
15- P.18 l.9: Please provide more details for the sequencing approach: read length, insert size, paired or single end?

**We added this information to the sequencing section.**
16- P.18 l.16: It might be good to specify that DESeq2 includes a library size normalization in its model to know that it has been done at some step of the analysis.

**Done.**
17- P.18 l.22-23: Some clarifications are needed regarding the normalization, is it to the maximum or minimum sample? Was this done before DESeq2?

**Clarifications have been added.**
18- P.19 l.11-15: What proportion of genes could be determined/annotated in this way? Could some of these categories just have 1 gene in them for example? Can it exaggerate the statistical significance? It is not clear how robust the method would be for custom gene sets determined that way.

**24,145 or 56% of genes were able to be annotated with the closest uniprot hit, and we have clarified this in the text. It is true that some of these gene sets have as little as 2 genes. However, the GSEA method considers how many genes are in the set in the p-value. For example, a set with just 1 gene could have a very high enrichment score if that gene is one of the top upregulated genes, but it would not be significant. Supplementary figure 2 and a comparing the gene set “size” column to the “padj” and “ES” (enrichment score) illustrates this relationship.**
19- P.19 l.15: More information on how the ranking is established and what the parameters mean would be helpful.

**We clarified what the “stat” parameter is and how/why we use it for ranking.**
20- Supp T1: not sure what the letters (S) (D) etc. mean, although I guess M for Missing etc.

**We have removed these for clarity (they are BUSCO’s abbreviation for each category but unnecessary here)**.
21- Supp T4: the gene corresponding to the dot labelled CNTN5 in fig1A seems to be named NPHN, are they the same?

**They are different annotations for the same gene. In some cases, the NCBI annotation (contactin-5-like – CNTN5) differs from the closest uniprot hit (NPHN), in which case we have to pick one to use in our labeling and discussion. Both are adhesion-related genes so they are likely closely related and would not change our interpretation of this result regardless of which we pick. For more systematic analyses, like GSEA gene sets, we only use the uniprot annotation.**
22- Supp T7: what is the agg_fold change?
**This stands for aggregate fold change and is a metric we used to rank these genes to enrich for those most up/down regulated in both the clam and mussel studies. We have changed this to mya_lfc*mtr_lfc to clarify that it is the log fold change from those two columns multiplied together.**

In the figures:
23- Figure 1: According to their description in the text, 60% genes (corresponding to ~26k genes) were not significantly up or downregulated. These are not visible in the plot. Please could this be clarified.

**These are difficult to see because of the log scale of the plot condenses those 60% of genes into the very bottom of the plot (0 - ~1.3: corresponding to adjusted p-value > 0.05) – a very small area relative to the size of the points. If you zoom in you can see there is a grey area corresponding to ~26k overlapping grey points. We have clarified this in the legend to avoid confusion for other readers.**
24- Figure 1 B (+) regulation of… means gene involved in positively regulating given pathway? Specify in legend.

**Done.**
25- Figure 1 and 4 need better annotating on the plot itself to understand what part of the plot is up and downregulated. In figure 4 add some labels directly on the plots to understand that A-B= conserved set of genes / C-D = MarBTN specific.

**We have added labels indicating which sample groups are being compared and directionality for both Figure 1 and 4, and titles to separate Fig4 into MarBTN-specific and conserved.**
26- For figure 2-B, it would be useful to plot the data as well as the boxes, the results seem very nice and convincing it would be great to see the actual data points that go with them. Also in the legend specify that we look at the median fold change (if it is the case).

**We have specified the Wilcoxon test compares medians in the legend. However, when we added points to 2B, we felt this complicated the interpretation since there are so many points per box plot (see N values at bottom of plot in the thousands, compare to smaller Ns in panels A and C: up to 200 and already getting crowded).**
27- Figure 3, add in legend that ASW= artificial sea water.

**Done.**
28- Supplementary figure 2: Define the red dotted line, and explain how the ranked list of genes is obtained.

**Done.**
29- Supplementary figure 3 and 5: although providing and appreciated control and being obviously well thought through, these figures still result a bit hard to read, some more annotation directly on the figure “quadrants” to make clear what is up and down regulated in what would be helpful.

**We added more labels with more complete explanations to the quadrants of these complicated figures.**
30- Supplementary figure 4: What exactly is the gene set in this figure? It looks like a fewer number than the entire set of genes, as seems to be stated in the legend.

**We can confirm this figure is for all expressed genes (~37k of the ~44k total genes) – perhaps the lower resolution of this image makes it look like less. We have sorted the genes to make groupings more obvious, as in supp fig 1, in response to another reviewer’s comments, and increased the resolution to make this figure more intuitive.**

Additionally, a few typos/reformulations:

**Thank you for catching these, they have been corrected.**
p.7 l.22 “Fusions found all cancer samples” – word “in” missing
p. 12 l 3-6: “The response to glucose/oxygen deprivation and negative regulation of apoptotic processes are among the top upregulated gene sets (Supplementary Table 14), indicating that these survival responses may be more broadly MarBTN-specific across additional genes.”: I am not sure I understand what the last part of the sentence means.
p. 19 l.13 “leaving excluding genes” - remove one verb.

Reviewer #3: This manuscript describes changes in gene expression in the Bivalve Transmissible Neoplasm from Mya arenaria, compared to its cell type of origin, a Hemocyte, and when it moves through an essential part of its life cycle, passage through seawater. As expected, the authors find vast number of genes significantly up and down regulated and highlight some of the most significant differences, which describe key aspects in the cancers ability to transmit. Interestingly, immune system pathways are down-regulated in MarBTN compared to the ancestral Hemocyte, indicating immune evasion. A subset of the gene expression changes are due to copy number variation (in an unstable genome) and the insertion of a retrotransposon, steamer. The most intriguing experiments for myself are the mimicry of sea water transmission and the plasticity in gene expression that allows a sedentary state for the cells. Overall, the paper is well written and clear and provides insights into cancer cell plasticity and transmission of interest across the cancer biology field. I have several queries and additions below:

• Page 8 – in the description of the genes up and down regulated it would be helpful for the reader if the log-fold change and p-value for each gene was provided in parentheses. It would give some context when read with Figure 1.

**We have added clarification, in response to another reviewer, for every gene and gene set whether it is up- or down-regulated when not explicitly stated. However, because there are so many genes and gene sets discussed in concordance with figure1a, 1b, 4a, 4b, 4c, and 4d, when we began to add labels with LFC and p-value throughout the paper we felt that it made the paper overly crowded with numbers and distracted from the content. If readers would like this information, they can see the approximate LFC and p-values in the graph and the exact values in the supplementary tables. In the one case in which genes are discussed that are not labeled in a main figure (when discussing Supplementary Fig 4 new results in response to comments below) we have added LFC and p-values).**
• Copy number variation is clearly playing a role in gene expression changes as the statistical tests and the number of DEGs show in 2B. What proportion of the DEGs does copy number variation explain?

**It is difficult to determine causation between copy number and expression for individual genes, further complicated by the polyploidy and complex copy number landscape of MarBTN. To address this and the following two comments we added a new analysis looking at the effect of CNV/Steamer/fusions on individual genes (Supp Fig 4). You can see that while some of the top genes are impacted by these mutation types, the majority are not, indicating that most differential expression is driven by other mutation types, epigenetic modifications, or indirect effects of mutated regulatory genes. We discuss a few of the top genes that may be directly affected by these mutation types and the context for other non-impacted genes in the text.**
• Similarly for the Steamer insertion, does steamer and CNV account for most of the expression change? This could be expressed in the results.

**See previous comment.**
• For the major genes identified in Figure 1, are these the result of CNV or steamer? If not, have the mechanisms been investigated?

**See previous comment.**
• The plasticity identified as the cells move into seawater is very interesting and could be explored further using the analysis on CNV and steamer above. For example, for the immune system genes de-regulated compared to Hemocytes and in sea water, are the mechanisms the same? There could be further explanation of this in the discussion.

**We agree this plastic response is very interesting and a key finding of our study. However, we would not expect the plasticity to be encoded by CNV mutations since cells in seawater or hosts would have the same copy number (copy number is steady across closely related samples, indicating it does not rapidly evolve: see our 2023 MarBTN genomics paper). It is possible that a transposable element like Steamer could drive this plasticity if it was up/downregulated in response to seawater, however we did not see evidence of this response in Steamer (see response to review 1 comment 5). We expect that the plasticity is driven by 1) inherent ability to survive in seawater as seen in hemocytes, and 2) further regulatory gene mutations or epigenetic changes, though it is difficult to elucidate which regulatory genes in particular may be driving this response. We have added discussion of these possibilities.**
• Line 13 page 13 – Join sentence to preceding paragraph

**This sentence has been expanded to present and discuss the results of the new analysis added in response to the comments above, so it has become a full paragraph.**
